# Time to Diagnosis in Dementia: A Systematic Review With Meta‐Analysis

**DOI:** 10.1002/gps.70129

**Published:** 2025-07-27

**Authors:** Olubunmi Kusoro, Moïse Roche, Rafael Del‐Pino‐Casado, Phuong Leung, Vasiliki Orgeta

**Affiliations:** ^1^ Faculty of Brain Sciences, Division of Psychiatry University College London London UK; ^2^ Faculty of Health Sciences Department of Nursing University of Jaén Andalusia Spain

**Keywords:** Alzheimer's disease, dementia, diagnosis, symptoms, time to diagnosis

## Abstract

Timely dementia diagnosis is a global priority, reflected in most national and regional policies and plans. Nevertheless, there are currently no robust estimates of the average time to diagnosis (TTD) and factors influencing diagnostic intervals. This article presents the first systematic review of quantitative studies on TTD in dementia and the factors associated with its duration. We systematically searched EMBASE, Psych INFO, MEDLINE, and CINAHL databases for relevant studies published up to December 2024. We defined TTD as the interval between symptom onset (rated by family carers or patients using interviews or medical records) to final diagnosis. Risk of bias was assessed using the Reporting studies on time to diagnosis tool. We included 13 studies reporting data on 30,257 participants, with age at onset ranging between 54 and 93 years. Meta‐analysis pooling 10 studies showed that average mean TTD across all types of dementia was 3.5 years [confidence interval (CI): 2.7–4.3; moderate quality evidence]. Analyses of six studies showed that TTD in young onset dementia was 4.1 years (CI: 3.4–4.9; moderate quality evidence). Although the factors influencing TTD were inconsistent, a younger age at onset and having frontotemporal dementia were consistently associated with a longer interval to diagnosis. TTD in dementia remains long, and specific healthcare strategies are urgently needed to improve it. Increasing the evidence base and developing interventions to reduce TTD should be a future research priority. Specialist services are likely to be key in improving TTD in young‐onset dementia.

## Key Points

- This study presents the first systematic review and meta‐analysis quantifying time to diagnosis (TTD) in dementia.
- Meta‐analytic data from 10 studies indicate an average TTD of 3.5 years across dementia types, with younger age at onset and frontotemporal dementia associated with longer diagnostic intervals.
- Interventions aimed at facilitating and shortening time to diagnosis in dementia are urgently needed.

## 1 Introduction

Dementia is a growing public health concern [1] with specific national and international strategies aimed at improving time to diagnosis (TTD) [2]. Despite a steady increase in the number of people affected by dementia, only 50%–65% of cases are diagnosed in high‐income countries [3], with rates much lower in low and middle‐income settings [4]. An increasingly recognised view is that a ‘timely diagnosis’ of dementia, is preferable to an ‘early diagnosis’ [5]. A timely diagnosis acknowledges that peoples' readiness to accessing treatment and care varies for both people with dementia and their families [6, 7, 8], and that decisions towards disclosure of diagnosis are influenced by individuals' preferences, circumstances and resources [9, 10, 11].

Despite the recent emphasis on timely diagnosis, quantitative data on the direct benefits for people with dementia and their family carers remain limited [7, 12]. Similarly, evidence reporting on the average TTD, and how this may differ across the different types of dementia remains small [13]. Dementia is a complex syndrome, making the recognition of the condition challenging for people with dementia, their families and healthcare providers [14, 15]. For a diagnosis to take place, an individual, or their family must first identify a problem, link that problem with the disease in question and decide to seek medical help [3]. In conditions such as dementia however, symptoms can often go unnoticed for several years [16, 17], with cognitive and functional changes often perceived as part of normal ageing or not severe enough to warrant treatment [18]. This often results in delays in help‐seeking and access to diagnosis [13, 19, 20].

TTD has been defined as the time from first alert of symptoms to the diagnosis of a disease [21]. In most western countries, General Practitioners (GP) refer patients to memory clinics when dementia is suspected, with referrals often occurring much later, long after the initial onset of symptoms [20]. Receiving a diagnosis of dementia is generally slow [14], with several condition‐specific and healthcare‐specific factors delaying the diagnostic process [19]. A dementia diagnosis is often disclosed long after symptoms are well established [22], with substantial delays specifically for young‐onset dementia [23]. Study estimates vary between 1 and 3 years [16, 24], with no systematic reviews identifying average TTD or the factors that influence an early or late TTD in dementia.

As a result the factors influencing TTD in dementia remain largely unknown [25, 26] despite these being systematically examined in other conditions [21]. This is problematic for wider initiatives aimed at facilitating a timely diagnosis, and informing future interventions. Identifying pathways from onset of symptoms to accessing care, and quantifying TTD for dementia is important for developing expedited pathways. Identifying key determinants both for longer or shorter TTD is key for informing future policy, improving help‐seeking behaviours and access to services such as diagnostic neuroimaging [26].

The aim of this study therefore was to conduct the first systematic review to quantify TTD in dementia defined as the time between symptom onset (as rated by patients or family carers) to receiving a diagnosis. A secondary objective was to identify the factors associated with an early or late TTD, and rate the quality of evidence to inform clinical guidelines, policy, and future research in the area.

## 2 Methods

This systematic review was conducted based on the guidelines of Preferred Reporting Items for Systematic Reviews and Meta‐Analyses [27], and was registered with PROSPERO (CRD42022372464). The titles, abstracts and full text of articles were screened independently by at least two reviewers (OK, MR, PL, VO, RdPC), and any disagreements were resolved with discussion.

### 2.1 Search Terms

We searched relevant terms such as ‘time to diagnosis’ and standardised dementia terms (see Supplementary materials) up to December 2024 in four major health databases (MEDLINE, EMBASE, PsycINFO, and CINAHL), including grey literature. We included additional terms, such as ‘assessment’, ‘referral pathway’, ‘memory clinic’, ‘delayed diagnosis’, ‘timely diagnosis’ and ‘time’ to ensure no relevant studies were missed. We also hand searched reference lists of identified articles.

### 2.2 Inclusion and Exclusion Criteria

We included articles of: (1) original retrospective or prospective cohort studies reporting on TTD, (2) in people who have received a formal diagnosis of any type of dementia based on established criteria (Diagnostic and Statistical Manual (DSM), International Classification of Diseases (ICD) criteria, National Institute of Neurological Disorders and Stroke (NINDS) criteria or comparable), (3) and those published in English. We excluded studies were: (a) dementia diagnosis was not based on clinical criteria or confirmed by medical records, (b) information on how onset of symptoms was measured was not specified, (c) those providing a narrative review of TTD, and (d) those reporting data based on surveys or case reports (see Supporting Information S1).

### 2.3 Data Extraction

Four review authors (OK, MR, PL, VO) independently extracted data, using a standardised data extraction form which was piloted before use. Data extracted included: study authors, study design, location, number of participants and demographic characteristics, details of the diagnostic pathway process, statistics quantifying TTD, confounders, and factors associated with longer or shorter TTD. Risk of bias was assessed independently by at least two reviewers using a revised version of the Reporting studies on time to diagnosis (REST) quality tool [21] addressing the following four domains: (1) *representativeness of the sample,* (2) *diagnosis* including a) clinical criteria for a dementia diagnosis, (b) blinding of reporting of diagnosis, (c) details of pathway, (3) *confounders*, and 4) *outcome and results* comprising (a) reporting of results, and b) details of number of participants lost to follow‐up.

### 2.4 Statistical Analysis and Synthesis of Results

We extracted data on intervals measured, specifying the beginning (symptom onset) and end time point (specialist consultation for final diagnosis and/or diagnosis). We performed a meta‐analysis pooling studies that provided adequate data (means and standard deviations), using a random effects model due to the variations in the population analysed (type of dementia and young onset vs. late onset). We calculated a pooled mean estimate and 95% confidence intervals (CI). All analyses were performed using the Comprehensive Meta‐analysis software 3.3 (Biostat Inc.). For those studies reporting medians, we asked for authors to provide us the value of the mean and standard deviation.

We measured statistical heterogeneity using the Q‐test and the degree of inconsistency (I^2^) to calculate the proportion of between‐study variability that was not due to chance. We assessed publication bias through the Egger's test, together with assessment of skewness in funnel plots, and applied the Trim and Fill method that estimates an effect size in a hypothetical case of no publication bias. We predicted a priori that TTD would differ by type of dementia and in young‐onset dementia, and therefore performed separate meta‐analyses on studies reporting on TTD in different types of dementia and those reporting estimates in young‐onset dementia.

## 3 Results

### 3.1 Study Results

**FIGURE 1:**
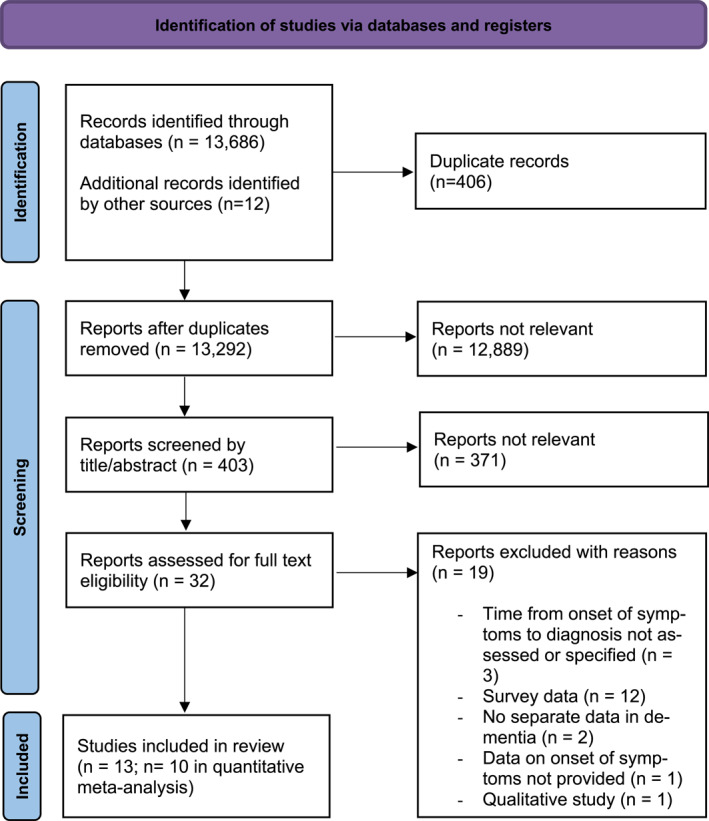
Flow chart of the search strategy.

A total of 13,686 articles were identified by the search (see Figure 1 for details of the search process), with 12 additional articles identified via hand‐search. After removing duplicates 13,292 articles remained, which were screened via title or abstract. After removing irrelevant articles, we screened 32 studies via full text for review eligibility. Of these, 19 were excluded with reasons (see Appendix in Supplementary materials) and 13 met inclusion criteria (see Table 1 for Characteristics of Included studies).

**TABLE 1:** Characteristics of included studies.

Study	Sample	Time to diagnosis definition, and/or diagnostic pathways assessed, and method of assessment of diagnosis	Time to diagnosis mean or median months or years (SD/range) or percentage	Predictors of time to diagnosis
Cattel (2000) Italy Referral period 1995–1996	– Patients presenting with cognitive deterioration referred to memory clinics living in the community *n* = 127 (*F* = 75; *M* = 52) Mean age: 73.9 (range 54–93 years) Mean MMSE: 15.3 Late– onset dementia: 84.3%; Young– onset dementia: 15.7% (no separate data) Dementia diagnosis (DSM– IV/NINCDS–ADRDA criteria): AD: 63%; Multi–infarct dementia: 26%; Other dementias: 11%	1. Time from onset of symptoms to visit for referral for neuropsychological testing and diagnosis 2. Onset of symptoms and referral for diagnosis assessed via semi– structured interviews with carers	Mean months = 13.8 (10.8)	Multivariate analyses Patients with ADL impairment (ADL ≥ 1) *Significant predictors of shorter TTD* – Lower MMSE (*β* = 0.31; *p* = 0.002) Patients with no ADL impairment *Significant predictors of longer TTD* – Increasing age (*β* = 0.50; *p* = 0.001) – Female sex (*β* = 0.51; *p* = 0.001) – Lower MMSE (*β* = −0.36; *p* = 0.017)
Chiari 2022 Italy Referral period 2017–2019	– Patients presenting with cognitive deterioration in a cognitive neurology clinic *n* = 168 (*F* = 100; *M* = 68) Mean age: 68.4 Mean MMSE: 22.0 Late–onset dementia: 56.5%; Young–onset dementia: 43.5% Dementia diagnosis (established clinical criteria i.e. McKhann et al. 2011): AD: 58.9%; FTD: 16.7% VAD: 8.3% LBD: 8.3% Other: 7.8%	1. a) Time from onset of symptoms to disease diagnosis b) Time from onset of symptoms to first assessment c) Time from onset of symptoms to diagnostic workup d) Time from onset of symptoms to dementia diagnosis 2. Onset of symptoms assessed via semi– structured interviews with carers and medical records	a) Mean months = 36.9 Mean months YOD = 41.8 Mean months LOD = 30.6 b) Mean months = 23.9 Mean months YOD = 28.4 Mean months LOD = 17.8 c) Mean months = 13.0 Mean months YOD = 13.2 Mean months LOD = 12.8 d) Mean months = 42.1 Mean months YOD = 45.5 Mean months LOD = 37.4	Univariate analyses *Significant predictors of longer TTD* – Age of onset (*β* = −0.256; *p* = 0.001) – YOD/LOD (*β* = 0.178; *p* = 0.022) – Time to first assessment (*β* = 0.670; *p* < 0.001) – Time of diagnostic workup (*β* = 0.737; *p* < 0.001)
Davis (2022) USA Referral period 1998–2013 Retrospective cohort	– Adult responders (non– Hispanic White and Non– Hispanic Black) (≥ 65 years) of the Health and Retirement study *n* = 3435* (*M* = 1293; *F* = 2142) Mean age: 81.6 *Reports on a subset of this sample: 30.1% (*n* = 1030) who received a diagnosis within 3 years of onset Dementia diagnosis: ADRDA and ICD–10 No details about dementia onset or type of dementia Dementia diagnosis (using ICD–10 & ICD–9 criteria identified by Medicare claims): No further details	1. Time from dementia onset identified by Telephone interview for cognitive Status (TICS) and date of healthcare Medicare claim 2. Onset of symptoms and referral for diagnosis assessed using TICS	% of sample that receives a diagnosis within 3 years of onset = 30.1% (1022 of 3435)	Multivariate analyses *Significant predictors of shorter TTD (defined as within 3 years of onset)* – Female sex (HR = 1.20 CI 1.03—1.39) – Having an additional ADL limitation (no data reported) *Significant predictors of longer TTD* *(defined as after 3 years of onset)* – High school educational attainment or less (HR = 0.65 CI 0.52–0.81) – Being a Black respondent (HR = 0.73 CI 0.61–0.88)
Draper (2016) Australia Referral period 2011–2012 INSPIRED study Retrospective cohort	– Adults presenting with dementia symptoms (≥ 65 years) referred by healthcare professionals, community organisations, or self–referred *n* = 88 (*M* = 63; *F* = 25) Mean age at onset: 55.4 Mean age at study interview: 62.6 Young– onset dementia diagnosis (DSM–IV/DSM– V criteria): AD: 53.4%; FTD: 15.9%; other causes: 30.7%	1. a) Time from onset of symptoms to dementia diagnosis b) Time from onset of symptoms to first consultation c) Time from onset of symptoms to family awareness of diagnosis d) Time from onset of symptoms to diagnosis of dementia type 2. Onset of symptoms assessed via structured interviews with patients and carers	a) Median years = 3.2 b) Median = 2.3 c) Median = 3.5 d) Median = 4.7	a) Not assessed b) Time from onset of symptoms to first consultation *Significant predictors of shorter TTD* – Younger age (HR 1.08 CI 1.04–1.12; *p* < 0.001) c) Time from onset of symptoms to family awareness of dementia diagnosis *Significant predictors of longer TTD* – Younger age (HR 1.10 CI 1.05–1.14; *p* < 0.001) – MCI diagnosis in pathway (HR 0.35 CI 0.19–0.66; *p* = 0.01) Other analyses Time from first dementia diagnosis to final dementia diagnosis *Significant predictors of shorter TTD* – Younger age (HR 0.94 CI 0.90–0.98; *p* = 0.01) – More years of education (HR 1.09 CI 1.03–1.17; *p* = 0.01) – Memory clinic as first specialist (HR 3.61 CI 1.35–9.65; *p* = 0.02) *Significant predictors of longer TTD* – MRI scans (HR 0.48 CI 0.26–0.91; *p* = 0.03) – FTD (HR 0.34 CI 0.17–0.67; *p* = 0.01) – Number of dementia types suggested (HR 0.43 CI 0.28–0.67; *p* < 0.001)
Kirson (2018) USA NACC UDS Referral period 2005–2015 Retrospective cohort	– Adults with suspected cognitive impairment referred by clinicians, family or community organisations *n* = 2950 (*M* = 1362; *F* = 1588) Mean age at diagnosis: 75.3 Mean MMSE: 21.8 No details about dementia onset Dementia diagnosis (NINCDS– ADRDA criteria): Probable AD (no further details)	1. Time from onset of cognitive decline to prAD diagnosis 2. Onset of symptoms assessed by interview	Median years = 4.5 (4.3 years for those diagnosed at baseline visit [*n* = 3493] and 5.0 years for those diagnosed after baseline visit [*n* = 935])	Analyses by early (within 3.5 years of cognitive impairment; *n* = 1476) versus late diagnosis (after 5.7 years; *n* = 1474) Baseline visit – Early diagnosis cohort was younger (*p* < 0.0001), less likely to be male (*p* = 0.0028), more likely to live alone (*p* < 0.0001), and less likely to have depression (*p* = 0.025) – Early diagnosis cohort had higher MMSE (p < 0.0001), lower CDR (*p* < 0.0001), higher FAQ (*p* < 0.0001), and lower NPI scores (*p* < 0.0001) Follow– up – Time to initiating AD– related medication was longer in the earlier diagnosis cohort (HR 0.91 CI 0.83–0.99; *p* = 0.027)
Koskas 2018 France Referral period 2006–2016 Retrospective cohort	– Community– dwelling older adults attending a memory clinic aged ≥ 65 years *n* = 1864 (*M* = 557; *F* = 1307) Mean age: 82.8 Mean MMSE: 17.9 No details about dementia onset Dementia diagnosis (DSM– IV/NINCDS–AIREN/McKeith/Lund– Manchester criteria): AD: 40.9%; VD: 12.2%; MD: 46.9%	1. Time between onset of signs and symptoms and referral (TOSR) for dementia to a memory centre 2. Onset of symptoms assessed by non– structured interview and self– administered questionnaire	Mean months AD = 40.8 (32.4) Mean months VD = 42.84 (38.6) Mean months MD = 37.68 (27.72)	Multivariate analyses (across wider sample including people with MCI *n* = 3353) *Significant predictors of longer TTD* – Increasing age (*p* < 0.0001) – Higher MMSE (*p* < 0.0001) – ‘Diagnosis of dementia not established’ (*p* < 0.0001)
Kvello– Alme 2021 Norway Referral period 2014–2018 Retrospective cohort	– Adults presenting with suspected cognitive impairment before the age of 65 in university hospitals *n* = 223 (*M* = 81; *F* = 142) Mean age at onset: 58.4 (range 47–64 years) Mean age at diagnosis: 63.3 (range 50–73) Mean MMSE: 23.0 Young– onset dementia Dementia diagnosis (NINCDS– ADRDA/McKhann criteria): AD	1. a) Time from onset of symptoms to diagnosis b) Time from onset of symptoms to contact with healthcare services (usually a GP) c) Time from contact with healthcare services (usually a GP) to first visit at a hospital d) Time from first visit at a hospital to diagnosis e) Time from first visit to hospital to analysis of cerebrospinal fluid core biomarkers 2. Onset of symptoms assessed via hospital notes, and interviews with carers	a) Mean years = 5.5 (2.8) b) Mean years = 3.4 (2.3) c) Mean months = 10.3 (15.5) d) Mean months = 14.8 (22.6) e) Mean months = 8.3 (20.9)	Not assessed
Leroy (2021) France Referral period 2010–2016 Retrospective cohort	– Community–dwelling outpatients with cognitive deterioration referred to memory clinics *n* = 19,521 (*M* = 5991; *F* = 13,530) Mean age at first visit: 73.7 Mean age at diagnosis: 74.4 Mean MMSE: 20.9 Late–onset and young– onset FTD: 71.8% versus 28.2% – total 3.5% No details for AD cohort—total 96.5% Dementia diagnosis (Rascovsky et al. 2011; Gorno–Tempini et al., 2011; Litvan et al., 1996; Armstrong et al., 2013 criteria)	1. a) Time from onset of symptoms to first referral to memory clinic network for diagnosis b) Time between first and last visit to the memory clinic network c) Time from first referral to last retained diagnosis 2. Onset of symptoms assessed by interviews with patients and carers	a) Mean months bvFTD = 40.0 (41.2) Mean lvFTD = 30.8 (20.5) Mean mFTD = 35.8 (29.9) Mean AD = 31.8 (32.0) b) Mean months bvFTD = 25.2 (24.0) Mean lvFTD = 24.2 (22.0) Mean mFTD = 20.2 (19.6) Mean AD = 17.5 (21.4) c) Mean months bvFTD = 9.9 (16.8) Mean lvFTD = 10.5 (16.4) Mean mFTD = 9.1 (15.1) Mean AD = 5.8 (14.2)	Predictors not assessed Comparisons between FTD and AD patients at first referral – FTD patients were younger (*p* < 0.0001), and more likely to be male (*p* < 0.0001) – FTD patients had higher MMSE (*p* < 0.0001), and IADL– 4 scores (*p* < 0.0001) – FTD patients were more likely to have a positive family history of dementia (*p* < 0.0001) – FTD patients had longer TTD (*p* < 0.0001), longer time from first referral to last retained diagnosis (*p* < 0.0001), and longer time from first and last visit to the memory clinic (*p* < 0.0001)
Loi (2022) Australia Referral period 2009–2019 Retrospective cohort	– Adults ≤ 65 years with behavioural, cognitive, and psychiatric symptoms referred to an inpatient specialist diagnostic unit *n* = 242 (*M* = 131; *F* = 111) Mean age at inpatient admission: 55.7 (range 24–70 years) Mean age of onset: 52.5 years (19–65 years) Mean NUCOG: 66.0 Young–onset dementia Dementia diagnosis (NINCDS–ADRDA/NIA– AA/Rascovksy criteria): AD: 30.6%; FTD: 29.3%; VaD: 11.1%; other dementia: 29%	1. Time between onset of symptoms to diagnosis 2. Onset of symptoms assessed by carers	Mean years = 3.4 (2.4) Median years = 2.0 (IQR = 2, 4; range = 0.5, 15 years)	*Significant predictors of longer TTD* – Younger age of onset (*β* = −0.21; *p* = 0.004) – Number of services consulted (*β* = 0.18; *p* = 0.013) – dementia diagnosis other than AD or bvFTD (*β* = 0.44; *p* = 0.011) *Differences in TTD between diagnostic groups* – AD and alcohol– related dementia had shorter time to diagnosis compared to ‘other dementia’ group (*p* = 0.02) *Effect of a specialist young– onset service* – Group accessing service had a shorter TTD compared to group that did not (*p* = 0.02)
Roberson (2005) USA Referral period 1988–2003 Retrospective cohort	– Outpatients presenting with cognitive impairment in a neurology clinic *n* = 572 (*M* = 243; *F* = 329) Mean age at onset FTD versus AD: 58.5 versus 70.1 Mean MMSE: 20.7 Late onset and young onset dementia Dementia diagnosis (Neary et al., (1998)/NINCDS– ADRDA/Litvan et al., 1996 criteria): FTD: 30.9%; AD: 69.1%	1. Time between symptom onset and initial evaluation 2. Onset of symptoms assessed by interviews with carers and patients	Mean years FTD = 4.5 (2.9) Mean years AD = 4.0 (2.8)	Predictors not assessed *Differences between diagnostic groups* – No differences in TTD between FTD and AD – Longer TTD in SD versus AD (*p* < 0.05)
Rosness 2008 Norway and Sweden Referral period not reported Retrospective cohort	– Outpatients < 65 years with cognitive deterioration assessed at memory clinics, neurology departments and hospitals *n* = 89 (*M* = 48; *F* = 41) Mean age at onset: 53.6 Mean age at diagnosis: 57.6 Mean MMSE: 21.0 Young–onset dementia Dementia diagnosis (Manchester–Lund and ICD– 10 criteria): FTD: 77.5%; AD: 22.5%	1. a) Time from onset of symptoms to diagnosis b) Time from first visit to medical doctor until specialist referral c) Time from specialist referral to correct diagnosis d) Time from first visit to medical doctor to correct diagnosis 2. Onset of symptoms assessed via patient records or interviews with carers	a) Mean months AD (Norway sample) = 39.1 (19.9) Mean months FTD (Norway sample) = 59.2 (36.1) Mean months FTD (Sweden sample) = 49.5 (24.5) b) Mean months AD = 19.1 (12.9) Mean months FTD = 23.8 (20.0) c) Mean months AD = 6.8 (5.1) Mean months FTD = 10.9 (12.8) d) Mean months AD = 25.9 (13.1) Mean months FTD = 34.5 (22.6)	Predictors not assessed *Differences between diagnostic groups* – Longer TTD in FTD versus AD (*p* < 0.01)
Van Vliet (2013) Netherlands Referral period not reported Two prospective cohorts	– Community–dwelling outpatients < 65 years referred from university medical centres, or enroled from memory clinics or healthcare services *n* = 402 (*M* = 194; *F* = 208) Mean age at diagnosis YOD versus LOD: 59.2 versus 78.6 Mean age at onset YOD versus LOD: 54.8 versus 75.8 Mean MMSE: 19.6 Late–onset dementia: 41.5%; young–onset dementia: 58.5% Dementia diagnosis (DSM– IV– TR/Dutch consensus guidelines criteria): AD: 64.9%; FTD: 8.0%; VD: 13.4%; MD: 3.5%; other: 10.2%	1. Time from onset of symptoms to diagnosis 2. Onset of symptoms assessed via patient records and semi– structured interviews with family carers and patients	Mean years = 3.8 (2.8) Mean years YOD = 4.4 (3.1) (range 0.5–18.0) Mean years LOD = 2.8 (2.1) (range 0.2–10.0) Mean AD = 3.6 (2.7) Mean FTD = 6.1 (3.5) Mean VD and MD*: = 3.1 (2.4) Other = 4.0 (3.0)	*Significant predictors of shorter TTD* – LOD as opposed to YOD (*β* = −0.20; *p* < 0.001) – VD and MD* as opposed to AD (*β* = −0.13; *p* < 0.05) *Significant predictors of longer TTD* – FTD as opposed to AD (*β* = 0.12; *p* < 0.05) *Differences in TTD between YOD and LOD groups* – YOD patients had longer TTD compared to LOD across all dementia types (*p* = 0.006)
Zhao 2015 China Referral period 2012–2013 Retrospective multicentre registry cohort	– Community–dwelling adults ≤ 45 years with suspected cognitive impairment recruited from memory clinics *n* = 576 (*M* = 267; *F* = 297; missing data = 12) Mean age at onset: 69.3 Mean age at study entry: 73.07 Late onset: 68.4%; young– onset: 30.7%; missing: 0.9% Dementia diagnosis: (Criteria not specified; use of Uniform data Set used in US AD research centres): AD: 68.2%; VD: 15.8%; FTD: 4.9%; other 11.1%	1. Time from onset of symptoms to medical visit seeking diagnosis 2. Onset of symptoms assessed by medical records and interviews with patients and carers	Median years = 1.77 Median months YOD = 22 (range 1–181) Mean months LOD = 21 (range 1–144) Median months AD = 24 (range 1–146) Median months VD = 9 (range 1–145) Median months FTD = 27 (range 5–67) Other types of dementia = 20 (range 1–181)	Multivariate analyses *Significant predictors of longer TTD* – Family history of dementia (*p* = 0.001) – Subtypes of dementia (*p* < 0.001); longest in FTD followed by AD and other dementias, and shortest for VD – Educational level (*p* = 0.03); less education increased TTD

Abbreviations: AD: Alzheimer's disease; ADL: activities of daily living; bvFTD: behavioural variant FTD; CDR: Clinical Dementia Rating; DSM– IV: Diagnostic and Statistical Manual of Mental Disorders, Fourth Edition; DSM– V: Diagnostic and Statistical Manual of Mental Disorders, Fifth Edition; DSM– IV– TR: Diagnostic and Statistical Manual of Mental Disorders, Fourth edition, text revision; F: Female; FAQ: Functional Assessment Questionnaire; FTD: Frontotemporal dementia; GP: General Practitioner; HR: Hazard ratio; IADL: Instrumental Activities of Daily Living; ICD– 9: International Classification of Diseases– 9; ICD– 10: International Classification of Diseases– 10; lvFTD: language variant FTD; NUCOG: Neuropsychiatry Unit Cognitive Assessment tool; M: Male; MCI: Mild Cognitive Impairment; MD: Mixed dementia; mFTD: motor variant FTD; MMSE: Mini– Mental State Examination; NACC UDS: National Alzheimer's Coordinating Centre Uniform Data Set; NIA– AA: National Institute on Ageing and Alzheimer's Association; NINCDS– AIREN: National Institute of Neurological Disorders and Stroke Association Internationale pour la Recherche et l'Enseignement en Neurosciences; NINDS– ADRD: National Institute of Neurological and Communicative Disorders and Stroke and the Alzheimer's Disease and Related Disorders Association; NPI: Neuropsychiatric Inventory Questionnaire; prAD: Probable AD; SD: Semantic dementia; VD: Vascular dementia.

*This group includes both vascular and mixed dementia.

### 3.2 Study Characteristics

#### 3.2.1 Study Samples

The 13 studies reported data of a total of 30,257 participants (with one study reporting data of only a subset of participants). Four studies investigated TTD specifically in young‐onset dementia [28, 29, 30, 31], three studies compared TTD between young versus late‐onset [32, 33, 34], with a further two studies investigating comparisons between AD and FTD [35, 36]. The remaining four studies investigated TTD in mixed samples (AD, VaD, other dementias) [37, 38], in probable AD [39], or did not specify dementia diagnosis [40]. There were seven European studies (Italy [34, 37], Netherlands [32], France [36, 38], Norway [29], Norway/Sweden [31]), two studies conducted in Australia [28, 30], three in the USA [35, 39, 40], and one study conducted in China [33].

Most studies reported data from cohorts recruiting outpatients from memory clinics, university hospitals, and neurology clinics, with one study taking place in an inpatient unit. Inclusion criteria were people presenting with symptoms of dementia referred by family members, community organisations, healthcare services or self‐referred. One study used data from Medicare claims [40].

#### 3.2.2 Pathways and Definitions of TTD

Table 2 provides details of TTD definitions and the specific pathways described across studies. Eight studies reported only one pathway to diagnosis [30, 32, 33, 35, 37, 38, 39, 40], with the remaining studies reporting additional pathways [28, 29, 31, 34, 36] which included the interval from onset of symptoms to first consultation, from first consultation to specialist referral, to additional pathways such as time from onset of symptoms to analysis of biomarkers (see Table 2).

**TABLE 2:** Pathways described across studies.

Study	*n* of pathways	Time to diagnosis definition and pathways described across studies	Reporting of TTD	Years to diagnosis
Cattel 2000	1	Time of onset of symptoms to date of visit for referral for neuropsychological testing and dementia diagnosis	Expressed in months—Mean and SD	Mean = 1.15
Chiari 2022	4	Time from onset of symptoms to disease diagnosis *Time from onset of symptoms to first assessment* *Time from onset of symptoms to diagnostic workup* *Time from onset of symptoms to dementia diagnosis*	Expressed in months—Mean and SD	Mean = 3.5
Davis 2022	1	Time from onset of symptoms assessed by telephone interview and referral for diagnosis	Expressed in months—Percentage that receives a diagnosis in 3 years	Reports on only 30% of the sample
Draper 2016	4	Time from onset of symptoms to diagnosis of dementia	Expressed in years—Median	Median = 3.2
		*Time from onset of symptoms to first consultation*		
		*Time from onset of symptoms to family awareness of diagnosis*		
		*Time from onset of symptoms to diagnosis of dementia type*		
Kirson 2018	1	Time of onset of cognitive decline to first prAD diagnosis	Expressed in years—Median	Median = 4.5
Koskas 2018	1	Time between onset of symptoms and referral for dementia to a memory centre	Expressed in months—Mean and SD	Mean = 3.4
Kvello‐Alme 2021	5	Time from onset of symptoms to diagnosis	Expressed in years—Mean and SD and range	Mean = 5.5
		*Time from onset of symptoms to contact with healthcare services (usually a GP)*	Expressed in years—Mean and SD and range	
		*Time from contact with healthcare services (usually a GP) to first visit at a hospital*	Expressed in months—Mean and SD and range	
		*Time from first visit at a hospital to diagnosis*	Expressed in months—Mean and SD and range	
		*Time from first visit to hospital to analysis of cerebrospinal fluid core biomarkers*	Expressed in months—Mean and SD and range	
Leroy 2021	3	Time from onset of symptoms to first referral to memory clinic network for diagnosis	Expressed in months—Mean and SD	Mean = 2.9
		*Time between first and last visit to the memory clinic*	Expressed in months—Mean and SD	
		*Time from first referral to last retained diagnosis*	Expressed in months—Mean and SD	
Loi 2020	1	Time between onset of symptoms to diagnosis	Expressed in years—Mean and SD	Mean = 3.4
Roberson 2005	1	Time between symptom onset and initial evaluation	Expressed in years—Mean and SD	Mean = 4.2
Rosness 2008	4	Time from onset of symptoms to diagnosis	Expressed in months—Mean and SD	Mean = 4.1
		*Time from first visit to medical doctor until specialist referral*		
		*Time from specialist referral to correct diagnosis*		
		*Time from first visit to medical doctor to correct diagnosis*		
Van Vliet 2013	1	Time from onset of symptoms to diagnosis	Expressed in years—Mean and SD and range	Mean = 3.8
Zhao 2015	1	Time from onset of symptoms to medical visit seeking diagnosis	Expressed in months and years—Median and range	Median = 1.8

#### 3.2.3 Meta‐Analyses on TTD

Pooling data from 10 studies (Table 3) showed that average mean diagnosis for all types of dementia was: 3.5 years (CI: 2.7, 4.3; *N* = 22,307; no heterogeneity; no critical asymmetry). The cumulative meta‐analysis by year of publication of studies shows that TTD has experienced a small increase over time (see Supplementary file Figure 3). We were able to conduct analyses by type of diagnosis; these showed that TTD for AD was 3.6 years (CI: 2.9, 4.3; 7 studies; *N* = 20,564; low heterogeneity; no critical asymmetry), and 4.2 years for FTD (CI: 3.2, 5.2; 5 studies; *N* = 1016; no heterogeneity; no critical asymmetry).

**TABLE 3:** Results of meta‐analyses.

			Average	Combined effect			Heterogeneity			Sensitivity		Publication bias			
					Lower	Upper				Analyses		Funnel	Egger's	Trim and Fill	
	*K*	*N*	*N*	*M*	Limit	Limit	Q (df)	*p*	*I* ^2^	*M* Max	%	Plot	*p*‐value	*M*	%
Dementia	10	22,307	22,307	3.52	2.68	4.35	6.12 (9)	0.73	0.0	3.77	7.1	Asym	0.09	3.52	0.0
AD	7	20,564	29,377	3.61	2.93	4.30	6.28 (6)	0.39	4.5	3.3	8.6	Asym	No val.	3.95	9.4
FTD	5	1016	203.2	4.21	3.19	5.23	4.40 (4)	0.35	9.1	3.80	9.7	Asym	No val.	4.21	0.0
YOD	6	972	162.0	4.13	3.37	4.88	3.46 (5)	0.63	0.0	3.83	7.3	Asym	No val.	4.28	3.6
YOD AD	4	454	113.5	3.97	2.71	5.23	2.45 (3)	0.48	0.0	3.45	13.1	Asym	No val.	3.97	0.0
YOD FTD	3	146	48.7	4.69	3.03	6.35	2.31 (2)	0.32	13.2	3.92	16.4	Sym	No val.	4.69	0.0
LOD	2	240	120.0	2.90	2.61	3.19	1.0 (1)	0.32	0.0	3.1	6.9	No val.	No val.	No val.	No val.

*Note:* Analyses overview: Dementia: all types; both young and late onset.

Abbreviations: %: percentage of variation from the original combined effect; AD: Alzheimer's disease, young onset and late onset; Asym: asymmetric; FTD: Frontotemporal dementia, young onset and late onset; K: number of studies; LOD: all types, only late onset; M: combined mean; M max: maximum value of the combined mean for sensitivity analysis eliminating one study at a time; N: sample size; No val: No value; Sym: symmetrical; YOD: all types, only young onset; YOD AD: Alzheimer's disease, only young onset; YOD FTD: Frontotemporal dementia, only young onset.

Pooling data from studies in young‐onset dementia showed that average TTD was 4.1 years (CI: 3.4, 4.9; 6 studies; *N* = 972; no heterogeneity; no critical asymmetry). Analyses by type of diagnosis showed that for young‐onset AD TTD was 4.0 years (CI: 2.7, 5.2; 4 studies; *N* = 454; no heterogeneity; no critical asymmetry) and 4.7 years for FTD (CI: 3.0–6.4; 3 studies; *N* = 146). Only two studies contributed on analyses for TTD in LOD, which showed average TTD was 2.9 years (CI: 2.6–3.2; *N* = 240). See Table 3 for details of all TTD analyses and Supplementary materials for forest plots and funnel plots.

### 3.3 Narrative Analysis of Factors Influencing TTD

#### 3.3.1 Studies on Mixed Samples, and Studies Not Specifying Dementia Diagnosis

##### 3.3.1.1 Predictors of Shorter TTD

In a single study, lower cognition predicted shorter TTD in people with impairment in activities of daily living (ADL) [37]. Female sex and having no ADL impairment also shortened TTD in a single study [40].

##### 3.3.1.2 Predictors of Longer TTD

Results were conflicting on cognition with one study reporting lower cognition increasing TTD [37], versus higher levels being predictive of longer intervals to diagnosis [38]. Two studies found that increasing age was associated with longer TTD [37, 38]. Female sex [37], and having no established diagnosis predicted longer TTD [38] in single studies. In one study not specifying type of dementia diagnosis, lower education and being Black increased interval to final diagnosis [40].

##### 3.3.1.3 Studies in AD and FTD

Findings were also inconsistent in relation to whether TTD differed between AD and FTD. In a single study TTD did not differ between AD and FTD [35], versus data from one study indicating longer intervals of diagnosis for FTD compared to AD [36], and longer intervals from first referral to last visit. In a single study [36], FTD patients were younger at diagnosis compared to those with AD, and more likely to have a positive family history of dementia. In a single study an earlier AD diagnosis was associated with lower levels of neuropsychiatric symptoms, and living alone [39], with time initiating medication longer at follow‐up [39].

#### 3.3.2 Studies in Young‐Onset Dementia

##### 3.3.2.1 Predictors of Shorter TTD

In a single study, having AD or alcohol‐related dementia, and accessing a specialist service were associated with shorter TTD [30].

##### 3.3.2.2 Predictors of Longer TTD

In two studies younger age was associated with longer TTD [28, 30]. Data from single studies showed that number of services consulted [30], a dementia diagnosis other than AD or bvFTD [30], and having an MCI diagnosis increased TTD [28]. Two studies reported that FTD was associated with longer intervals to final diagnosis [28, 31].

### 3.4 Studies Comparing Young and Late‐Onset Dementia

Three Studies Compared TTD in People With Young and Late‐Onset Dementia [32, 33, 34].

#### 3.4.1 Predictors of Shorter TTD

LOD and having mixed dementia were associated with shorter TTD in a single study [32], and having vascular dementia predicted a shorter diagnostic interval in two studies [32, 33].

#### 3.4.2 Predictors of Longer TTD

Data from two studies showed that having YOD [32, 34] and FTD [32, 33] increased TTD. In single studies age of onset, time to first assessment, time to diagnostic workup [34], less education and having a family history of dementia [33] increased the diagnostic interval.

### 3.5 Quality of Studies

Two reviewers assessed quality of studies independently (see Table 2, Supplementary materials). Overall samples were representative of people with dementia, with clinical criteria adequately described. Many of the studies however did not adequately control for confounders that could be influencing TTD, and detailed pathways to diagnosis were not always described [21]. Low quality was also observed in reporting of statistics. Full details of quality ratings across all studies meeting inclusion criteria are provided in Supplementary materials.

## 4 Discussion

Our study is the first systematic review and meta‐analysis reporting on evidence examining TTD in dementia. We found that TTD in dementia remains long and is on average over 3.5 years. We identified a small evidence base overall, with a total of 13 studies conducted to date, indicative of limited quantitative evidence [12]. An important strength of our study is that we were able to provide the first quantitative estimate of TTD in dementia pooling global evidence. Although delayed diagnosis to some extent is understandable, our results indicate that TTD in dementia remains long. For example, in many of the studies reporting pathways beyond first contact with healthcare services, delays receiving a diagnosis were still observed, even in the context of specialist clinics [28, 30]. We also found that TTD varied across diagnostic groups, with consistent evidence of longer TTD in young‐onset dementia [29], a finding that was observed across countries.

Our review is the first to highlight the lack of standardized criteria for measuring TTD in dementia [12], and the observed heterogeneity across studies such as variations in sample size, and different definitions of TTD [13]. Although this heterogeneity may result in future large‐scale studies providing a different estimate, our meta‐analysis was based on moderate quality studies overall, which means we can be relatively confident that the true estimate is close to the one reported in our review.

An important limitation of current research is that factors that influence TTD in dementia were based on findings from single studies [11]. This limits any recommendations for policy and healthcare initiatives that could be implemented to shorten TTD in dementia [5, 12]. Below we discuss findings on factors identified that influence TTD.

### 4.1 Factors Affecting TTD in Studies of Both Late‐ and Young‐Onset Dementia

Data from one study showed that impairment in activities of daily living (ADL) influences TTD, with lower cognitive function being associated with shorter TTD in those experiencing functional decline [37]. On the contrary, in those with no ADL impairment, lower cognition, increasing age and being female was associated with longer TTD [37]. These results suggest that people who experience functional decline, may be referred to services earlier, and that the more severe the deterioration, the earlier the referral [37]. We found evidence from two studies that younger age of onset was associated with longer TTD [34], across both young and late‐onset dementia, which is in line with prior work [41]. However, not all studies reported similar effects on the influence of age, female sex, and levels of education [40]. It will be important that future large‐scale studies are conducted that investigate which specific factors influence TTD across young and late‐onset dementia [32].

Only one study [40] examined the effect of race on TTD. Although this study only reported data of a subset of participants, specifically those receiving a diagnosis within 3 years to onset, it found that Non‐Hispanic Black individuals had a longer TTD compared to Non‐Hispanic Whites, with differences partially explained by income and educational attainment [40]. This preliminary finding is consistent with well‐known ethnic disparities of accessing a dementia diagnosis [42, 43], including diagnostic neuroimaging services [26]. From a policy perspective, our findings implicate broader race‐associated gaps within the healthcare system that may impact long‐term outcomes and care choices [44].

In the study by Kirson 2018 those receiving an early diagnosis had higher cognition and function, but time to initiating AD‐related medication was longer compared to those receiving a later diagnosis (5.7 years after cognitive impairment onset). In the study by Zhao 2016, a family history of dementia and lower education increased TTD. Although these findings remain preliminary, they suggest that several factors influence TTD, therefore increasing the evidence base of parameters influencing TTD is key in developing interventions to facilitate a timely diagnosis [13].

### 4.2 Factors Affecting TTD in Studies of Young‐Onset Dementia

For young‐onset dementia, younger age were associated both with longer TTD from onset of symptoms to final consultation and family awareness of dementia [28]. The same study reported that an MCI diagnosis within the diagnostic pathway further delayed TTD [28]. Having FTD, use of MRI and number of dementia types suggested, also increased the interval to final diagnosis [28]. These results suggest that the factors that contribute to the time until a final diagnosis is made vary according to the stage of the diagnostic process in young‐onset dementia. For example, in the same study, being of younger age, having more years of education and attending a specialist memory clinic, shortened time from first to final diagnosis. Although preliminary our findings are in line with previous studies indicating that access to specialist services will be key in shortening TTD in young‐onset dementia [41].

### 4.3 Type of Dementia and TTD

Evidence from several studies showed that receiving a diagnosis of FTD is associated with longer time to final diagnosis [31, 32]. These findings are important as they suggest that FTD syndromes take longer to diagnose overall even within regions with organised memory clinic networks, despite novel clinical criteria and incorporation of new phenotypes [45]. It should be noted however that not all studies reported longer TTD for FTD; it may be for example that TTD differs across healthcare settings and specialist clinics [18, 46]. Future large‐scale studies are needed to address differences across different types of dementia and the factors that influence TTD which could be specific to each dementia type [13, 46]. These studies should also evaluate the effectiveness of specialist training for clinicians on shortening TTD over time.

Two studies identified in our review reported that vascular dementia is overall associated with a shorter TTD compared to AD and FTD [32, 33]. One potential explanation may be that symptoms of vascular dementia develop acutely with the onset of cerebrovascular events [47]. Although data remain limited these findings indicate that this form of dementia is diagnosed early and mechanisms in place that assist with this may inform diagnostic pathways for other types of dementia [48].

### 4.4 Clinical Implications

An important finding of our review is that despite the emphasis towards timely diagnosis and world‐wide initiatives, dementia diagnosis remains very long, especially for people with young‐onset dementia, who continue to face a significant delay in diagnosis globally [9, 41]. Our review highlights the need for a conceptual framework, that encompasses a consensus based upon scientific evidence on acceptable intervals and TTD in dementia [13]. The diagnostic interval conceived as the time between first consultation with a health professional and a definitive diagnosis was not investigated in many of the studies and should be a priority of future investigations.

Our findings provide preliminary evidence that establishing specialist centres for people with young‐onset dementia may shorten the length of diagnostic delay for this group [30]. This is consistent with evidence that such specialist services may increase the numbers of people with dementia assessed nationally, which could then lead to quality of life improvements [49].

Future research should harmonise definitions of TTD and the different stages of the diagnostic process, including diagnostic experiences [48, 50]. Improving TTD is likely to be key for earlier intervention, and reducing both the economic and societal costs of dementia [51]. Future large scale quantitative studies are needed to understand how TTD influences patient and carer outcomes [5, 12], and establishing the potential benefits or harms of a timely diagnosis [12].

### 4.5 Limitations

An important limitation of our review and meta‐analysis is that definitions of TTD differed across studies, with different methods being used to assess the first symptoms of dementia, which may have influenced results. We were also not able to meta‐analyse data on factors influencing TTD. Some studies used questionnaires to retrospectively assess first symptoms, whereas other studies used clinicians' reports during baseline clinical visits. Many of the studies did not control for recall bias, and it is likely that people experiencing severe dementia may not have accurately remembered when they first experienced symptoms. This means that our meta‐analysis may have used rather strict (with a shorter TTD) or more permissive (longer TTD) definitions. Our findings are unlikely to be applicable to low and medium‐income countries (LMICs), given we found no studies in these settings, and that we included only studies published in English. Future studies assessing health system factors influencing TTD should be a priority, as well as investigating diagnostic delays due to COVID‐19 [52].

Our review highlights that although delays in TTD in dementia are influenced by the complexity of symptoms, the diagnosis process itself within healthcare services remains long. Designing prospective, population‐based studies controlling for potential confounding variables is strongly recommended together with adherence to standardised guidelines on TTD. Further research is needed to examine differences between countries and settings with higher versus lower investments in healthcare systems. To increase the effectiveness of community‐based awareness campaigns, interventions should focus on identifying and targeting high‐risk groups and adapt these to the population's socio‐cultural context.

## 5 Conclusion

This review is the first to systematically assess and quantify evidence base of TTD in dementia. Our findings have significant implications for future policy, practice and research relevant to many countries. To increase effectiveness of future interventions, it is paramount to identify which socio‐demographic, disease specific, and healthcare specific factors contribute to a longer TTD in dementia. Development of interventions that aim to reduce the diagnostic interval in dementia are urgently needed.

## Author Contributions

**Olubunmi Kusoro:** conceptualisation of the review, selection of studies, data extraction, data analysis, data quality, writing, review and editing of manuscript. **Moïse Roche:** conceptualisation of the review, selection of studies, data extraction, data analysis, data quality, review and editing of final draft. **Rafael Del‐Pino‐Casado:** conceptualisation of the review, selection of studies, data extraction, data analysis, review and editing of final draft. **Phuong Leung:** conceptualisation of the review, selection of studies, data extraction, data analysis, review and editing of final draft. **Vasiliki Orgeta:** conceptualisation of the review, selection of studies, data extraction, data analysis, data quality, writing, review and editing of manuscript.

## Conflicts of Interest

The authors declare no conflicts of interest.

## Supporting information

Supporting Information S1

## Data Availability

The data that support the findings of this study are available on request from the corresponding author (VO); please email v.orgeta@ucl.ac.uk.
