# Supplementary material for: Time to Diagnosis in Dementia: A Systematic Review With Meta‐Analysis
Source: Int J Geriatr Psychiatry. 2025 Jul 27;40(7):e70129. doi: 10.1002/gps.70129 (PMC12300619; doi:10.1002/gps.70129)

Figure 1. Search strategy of the review (this included both MeSH and keyword terms)

1. “assessment”/ or “referral pathway”/ or “memory clinic”/ “delayed diagnosis”/ “timely diagnosis”/
2. assessment* or referral pathway* or memory clinic* or delayed diagnosis* or timely diagnosis*
3. “dementia”/ or” Alzheimer’s disease”/ or “late onset” / or “early onset” / or “young onset”/ or “time”/ or “onset”
4. dementia* or Alzheimer’s disease* or late onset* or early onset* or young onset* or time* or onset*
5. 1 or 2
6. 3 or 4
7. 5 and 6

Table 1. Excluded studies with reasons

| Study | Reason for exclusion |
| --- | --- |
| Ajnakina 2020 | Retrospective cohort study reporting TTD but no specific data on onset of symptoms |
| Black 2019 | Survey reporting on physicians’ views on delays of TTD |
| Boise 1999 | Survey of family carers on factors which delayed obtaining a diagnosis |
| Calvó-Perxas 2012 | Cross-sectional study of TTD; no specific details of measurement of onset of symptoms |
| Chen 2019 | Survey data based on Medicare claims reporting on percentage of people diagnosed > 2 years or <2 years after  onset of cognitive decline; no formal diagnosis of dementia |
| Chrisp 2011 | Survey assessing time of onset of symptoms to visiting a memory clinic; TTD not specified |
| de Miranda 2011 | Cross-sectional study of carers’ perceptions of delays of TTD; no data on TTD |
| Gély-Nargeot 2003 | Survey of carers of people with AD on current practice and disclosure of diagnosis |
| Helvik 2018 | Cross-sectional study reporting on time from symptom onset to dementia consultation; no separate data for people with dementia |
| Kumagai 2024 | Survey on TTD as reported by carers |
| Luscombe 1998 | Survey reporting on problems to diagnosis experienced by family carers of people with young-onset dementia |
| Ritchie 2018 | Survey on time of symptom onset, referral and subsequent diagnosis based on physician data |
| Schrauf 2011 | Qualitative study using card sort interviews to identify pathways of delayed diagnosis |
| Speechly 2008 | Survey on pathways to diagnosis as reported by carers |
| Tsoukra 2022 | Retrospective study on diagnostic challenges; no separate data on people not experiencing a change in diagnosis |
| Wilkinson 2004 | Survey on access to diagnosis and care |
| Williams 2001 | Cross-sectional study reporting on referral pathways for people with young-onset dementia; no data on TTD |
| Wolf 2020 | Survey on TTD as reported by carers |
| Woods 2018 | Survey on TTD as reported by carers |

Table 2. Quality of included studies using the Reporting Studies on Time to Diagnosis (REST) tool

| Study | Representativeness of the sample | Clinical criteria for dementia diagnosis | Blindness of reporting of diagnosis | Details of pathway | Confounders  assessed | Reporting of statistics | Number of participants lost and reasons | Quality |
| --- | --- | --- | --- | --- | --- | --- | --- | --- |
| Cattel 2000 | 1 | 1 | 0 | 1 | 2 | 2 | 1 | Moderate |
| Chiari 2022 | 1 | 1 | 0 | 1 | 2 | 2 | 1 | Moderate |
| Davis 2022 | 1 | 1 | 0 | 1 | 2 | 0 | 0 | Poor |
| Draper 2016 | 1 | 1 | 0 | 2 | 2 | 1 | 1 | Moderate |
| Kirson 2018 | 2 | 1 | 0 | 1 | 2 | 1 | 2 | Moderate |
| Koskas 2018 | 2 | 1 | 0 | 1 | 2 | 2 | 1 | Moderate |
| Kvello-Alme 2021 | 1 | 1 | 0 | 2 | 0 | 2 | 1 | Moderate |
| Leroy 2021 | 2 | 1 | 0 | 2 | 0 | 2 | 1 | Moderate |
| Loi 2020 | 1 | 1 | 0 | 1 | 2 | 2 | 1 | Moderate |
| Robertson 2005 | 1 | 1 | 0 | 1 | 0 | 2 | 1 | Moderate |
| Rosness 2008 | 1 | 1 | 0 | 2 | 0 | 1 | 1 | Moderate |
| Van Vliet 2013 | 1 | 1 | 0 | 1 | 2 | 2 | 1 | Moderate |
| Zhao 2015 | 1 | 1 | 0 | 1 | 2 | 2 | 1 | Moderate |

Figure 2. Forest plot for Time to diagnosis in all types of dementia


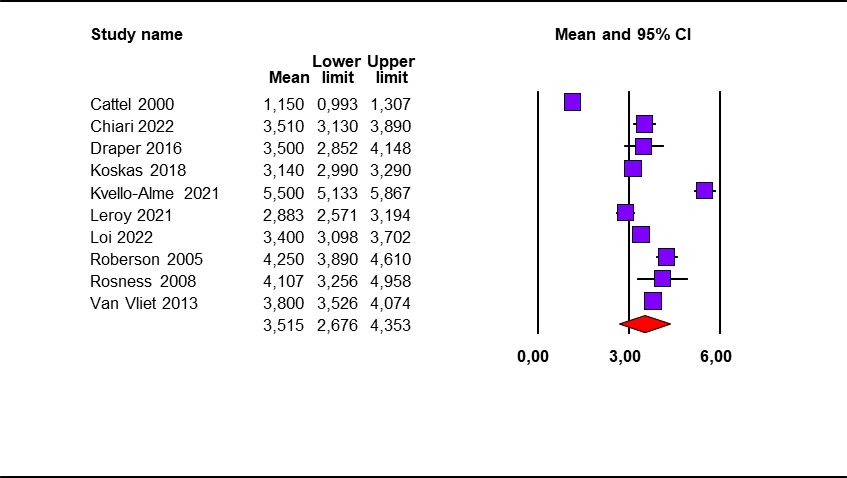


Figure 3. Cumulative forest plot for Time to diagnosis in all types of dementia (studies by year of publication –from oldest to newest)


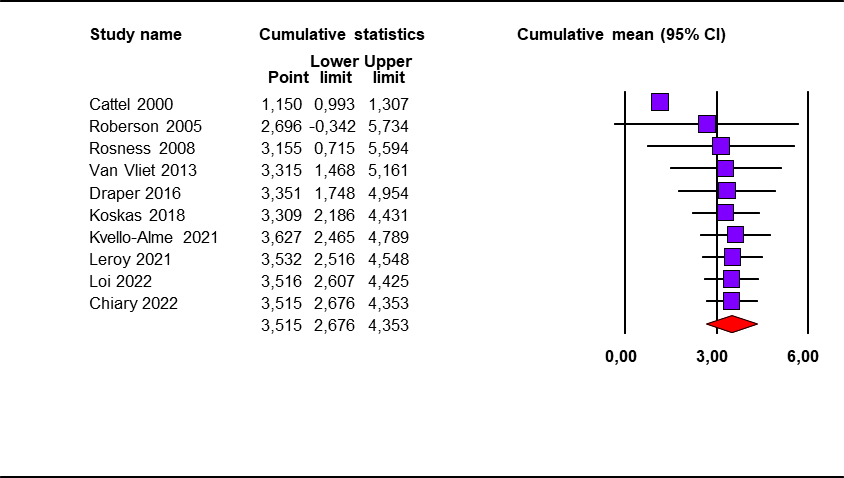


Figure 4. Funnel plot for Time to diagnosis in all types of dementia

Figure 5. Forest plot for Time to diagnosis in Alzheimer’s disease (young and late onset)


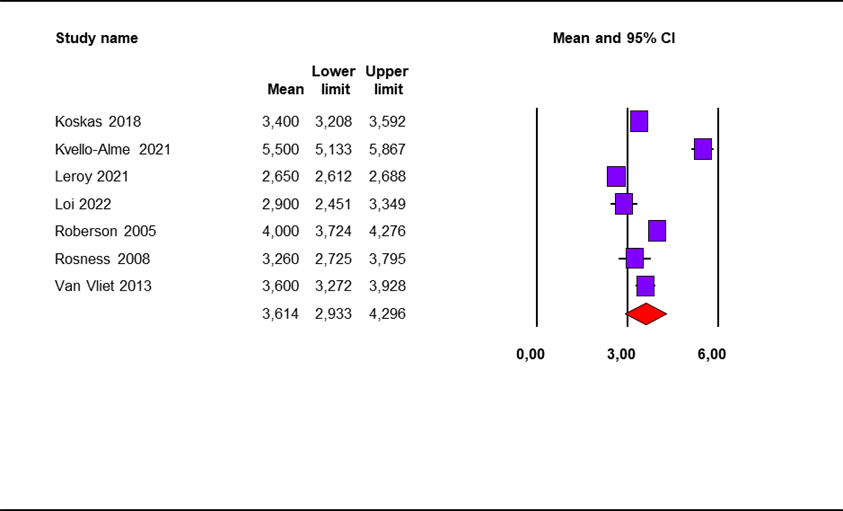


Figure 6: Funnel plot for Time to diagnosis in Alzheimer’s disease (young and late onset)

Figure 7. Forest plot for Time to diagnosis in Frontotemporal dementia (young and late onset)


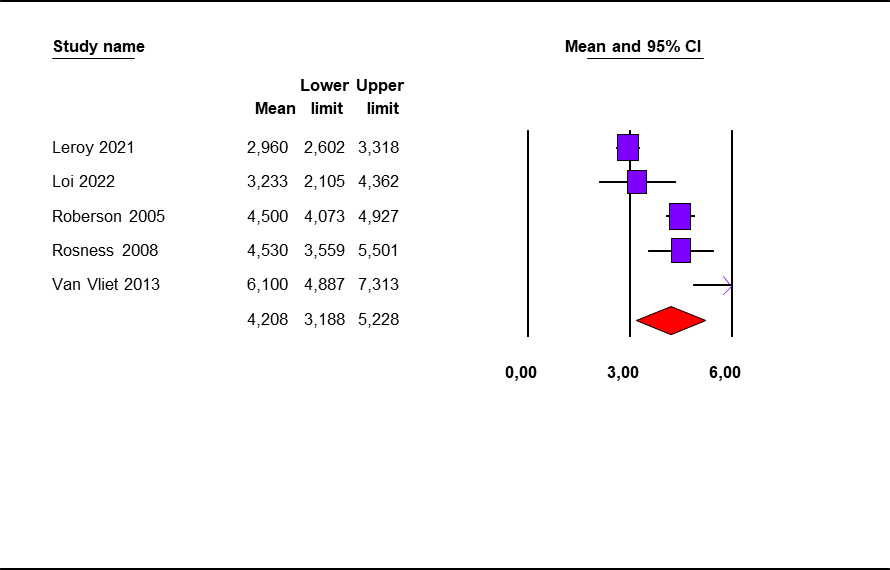


Figure 8. Funnel plot for Time to diagnosis in Frontotemporal dementia (young and late onset)


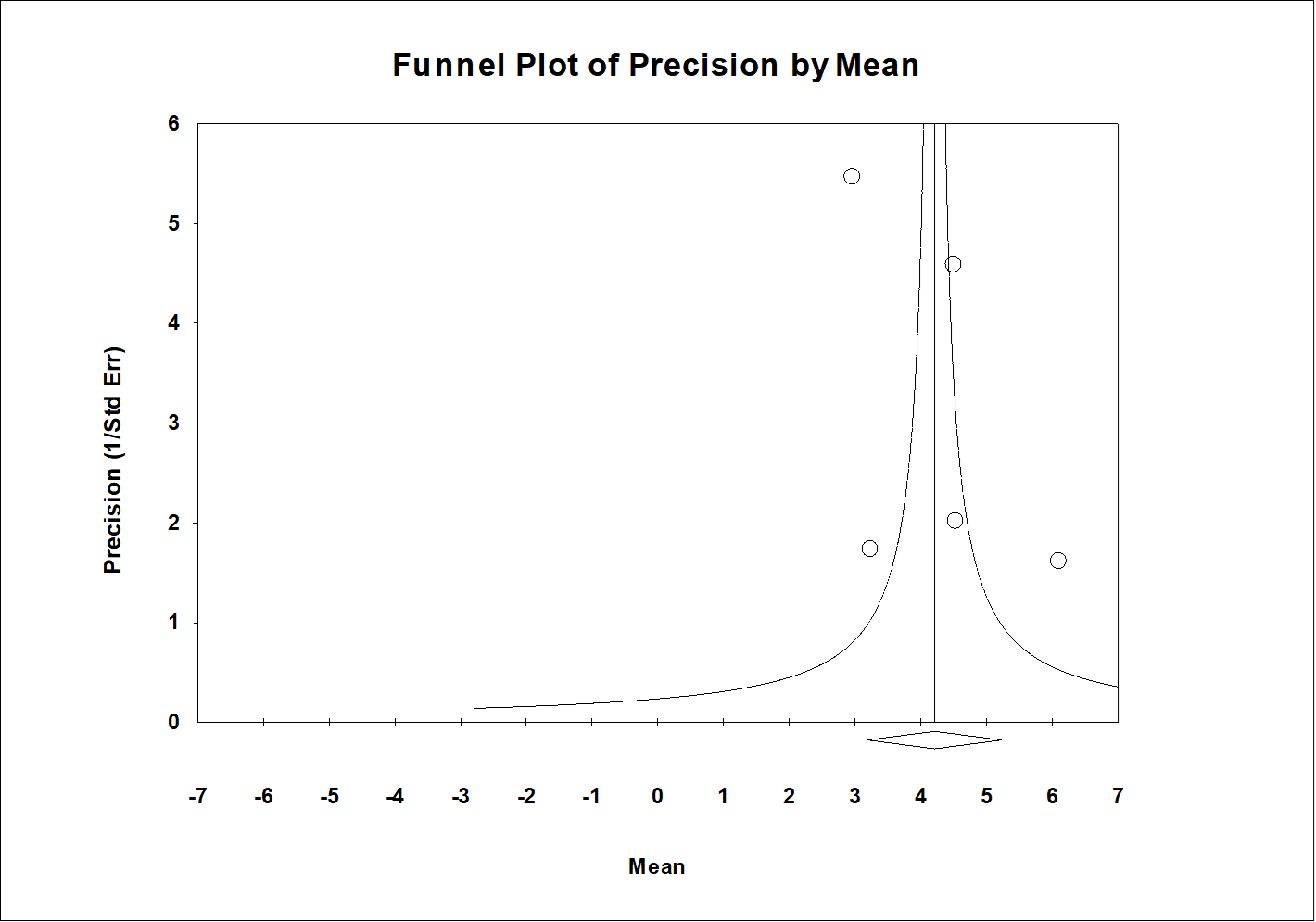


Figure 9. Forest plot for Time to diagnosis in Young onset dementia (all types of dementia)


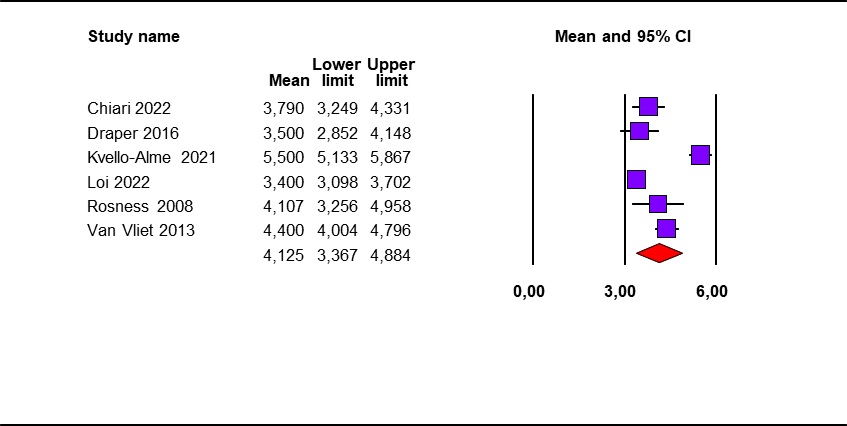


Figure 10. Funnel plot for Time to diagnosis in Young onset dementia (all types of dementia)

Figure 11. Forest plot for Time to diagnosis in Alzheimer’s disease, young onset


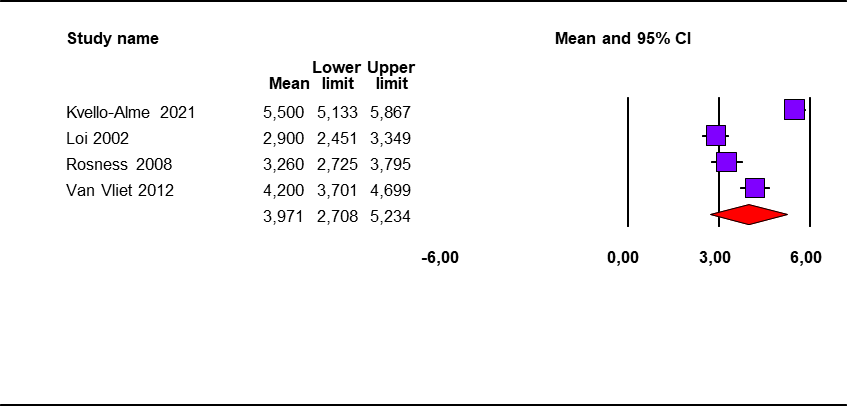


Figure 12. Funnel plot for Time to diagnosis in Alzheimer’s disease, young onset

Figure 13. Forest plot for Time to diagnosis in Frontotemporal dementia, young onset


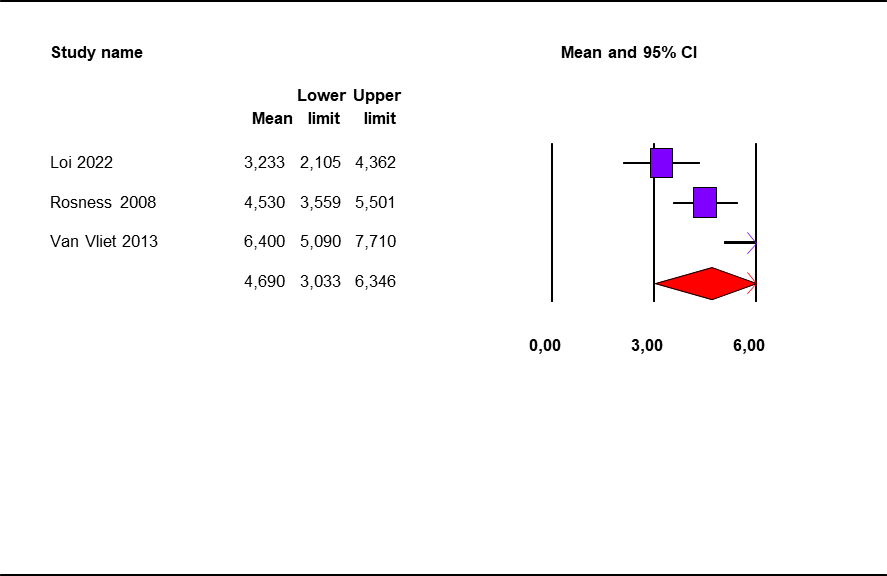


Figure 14. Funnel plot for Time to diagnosis in Frontotemporal dementia, young onset

Figure 15. Forest plot for Time to diagnosis in Late onset dementia (all types of dementia)


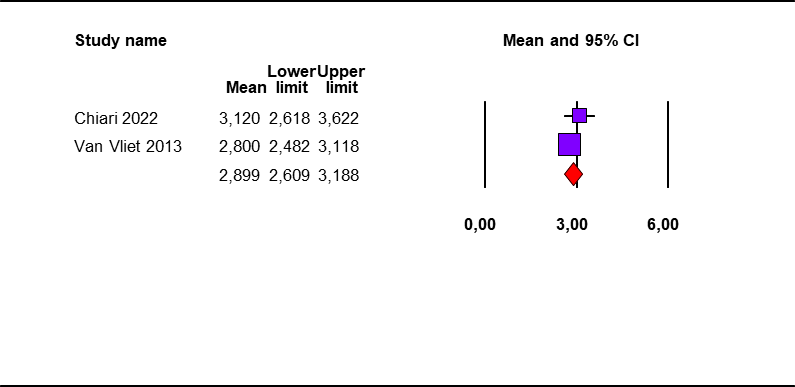

Supplement: Supplementary file 1 — Supporting Information S1 [file GPS-40-e70129-s001.doc]
